# Assembly and analytical validation of a metagenomic reference catalog of human gut microbiota based on co-barcoding sequencing

**DOI:** 10.3389/fmicb.2023.1145315

**Published:** 2023-05-05

**Authors:** Yufen Huang, Puzi Jiang, Zhengjiao Liang, Rouxi Chen, Zhen Yue, Xuefeng Xie, Changge Guan, Xiaodong Fang

**Affiliations:** ^1^BGI Genomics, BGI-Shenzhen, Shenzhen, China; ^2^BGI-Shenzhen, Shenzhen, China; ^3^BGI-Sanya, BGI-Shenzhen, Sanya, China; ^4^State Key Laboratory of Dampness Syndrome of Chinese Medicine, The Second Affiliated Hospital of Guangzhou University of Chinese Medicine, Guangzhou, China

**Keywords:** metagenomic, stLFR, single nucleotide polymorphism (SNPs), horizontal gene transfer (HGT), insertions/deletions (INDELs)

## Abstract

Human gut microbiota is associated with human health and disease, and is known to have the second-largest genome in the human body. The microbiota genome is important for their functions and metabolites; however, accurate genomic access to the microbiota of the human gut is hindered due to the difficulty of cultivating and the shortcomings of sequencing technology. Therefore, we applied the stLFR library construction method to assemble the microbiota genomes and demonstrated that assembly property outperformed standard metagenome sequencing. Using the assembled genomes as references, SNP, INDEL, and HGT gene analyses were performed. The results demonstrated significant differences in the number of SNPs and INDELs among different individuals. The individual displayed a unique species variation spectrum, and the similarity of strains within individuals decreased over time. In addition, the coverage depth analysis of the stLFR method shows that a sequencing depth of 60X is sufficient for SNP calling. HGT analysis revealed that the genes involved in replication, recombination and repair, mobilome prophages, and transposons were the most transferred genes among different bacterial species in individuals. A preliminary framework for human gut microbiome studies was established using the stLFR library construction method.

## Introduction

The human gut microbiota is the most diverse among all human microbiomes (Yahara et al., 2021), and it harbors hundreds of coexisting bacteria. This ecosystem plays a vital role in human health through various physiological processes, such as fermenting non-digestible dietary fiber, anaerobic metabolism of peptides and proteins, and immune system modulation (Guarner and Malagelada, 2003; Rooks and Garrett, 2016; Cai et al., 2022). Therefore, extensive research has been carried out on this subject, and the Human Microbiome Project (HMP) was proposed to further our understanding of gut microbiota (The Human Microbiome Project Consortium, 2012). The gut microbiota has been linked to several human diseases, such as diabetes, colon cancer, and inflammatory bowel diseases (Guarner and Malagelada, 2003; Mokkala et al., 2021; Tierney et al., 2021), and it can also affect the human brain through the gut–brain axis (Fairbrass et al., 2022). Despite extensive research, our current understanding of this complex ecosystem based on existing technologies is still inadequate.

Recently, the main technical approaches for studying gut microbiota involve sequencing methods and metabolomics (Arnold et al., 2016). Based on high-throughput and low-cost next-generation sequencing (NGS), the 16S rRNA of bacteria is commonly used for species diversity and abundance studies (Cho and Blaser, 2012). However, most gut microbes are difficult to cultivate, and the function and genome of the target strain cannot be analyzed and understood. To obtain the genome of a single gut microbe, metagenome sequencing, and corresponding analytical methods have been applied to recover bacterial genomes and characterize their functions (Hugenholtz and Tyson, 2008). To improve the accuracy of the binning step in the metagenome analysis process for obtaining high-quality genome assemblies, many bioinformatics software tools have been developed (Wu and Ye, 2011; Mande et al., 2012; Breitwieser et al., 2019). Nevertheless, the accuracy of these methods is still unsatisfactory due to technical problems such as short sequencing read lengths.

Despite some progress, the assembly of gut microbial genomes still has limitations. These limitations include the absence of reference genomes, poor assembly quality in next-generation sequencing (NGS), and difficulty in determining the positional relationship between genes. In particular, the use of metagenomic linkage groups (MLGs) as a replacement for true linkage relationships further hinders the assembly process. Additionally, poor genome assembly results in a lack of strain information, which ignores intraspecific diversity and makes it challenging to identify differences between different strains within a species and individual strains (Niccum et al., 2020). This difficulty in accurately analyzing differences between strain genomes also makes it challenging to accurately capture horizontal gene transfer (HGT) information, which is necessary for understanding the gut microbial community (Brito, 2021).

To address the aforementioned issues, third-generation sequencing (TGS) technologies were introduced to generate complete genomes from microbial communities (Chin et al., 2013; Bertrand et al., 2019; Kolmogorov et al., 2020). However, the high sequencing error rates in TGS hinder the distinction between true variants and sequencing errors. Co-barcoding sequencing library is an improved short-read sequencing technology with long-range genomic information (Peters et al., 2012; Adey et al., 2014; Bishara et al., 2018; Wang et al., 2019; Chen et al., 2020), which provides an alternative way to accurately and quantitatively analyze metagenomes. The total barcode number and the short-read coverage of HMWs have a great impact on the effectiveness of different co-barcoding libraries, such as BGI's stLFR library, 10X Genomics' linked-reads library, and Illumina's contiguity preserving transposase sequencing library, in downstream analysis. The co-barcoding correlation between assembled draft sequences and barcode distribution on the assembled graph has been successfully applied to both single genome and metagenome assembly (Chen et al., 2020; Roodgar et al., 2021; Kong et al., 2022; Siranosian et al., 2022).

This study aimed to explore the potential of the stLFR library method for application in metagenomics and compare it with the standard metagenomic library method to evaluate whether the stLFR library method can address issues of poor assembly results in standard second-generation sequencing. To achieve this, we applied the stLFR method to the metagenomic assembly of a Chinese population fecal sample containing 21 individuals and constructed a reference microbial genome for further analysis of the microbiome genome. We performed SNP, INDEL, and HGT gene analyses to demonstrate the effectiveness of the reference genome assembled using the stLFR method.

## Method

### Sample collection

**Figure 1:**
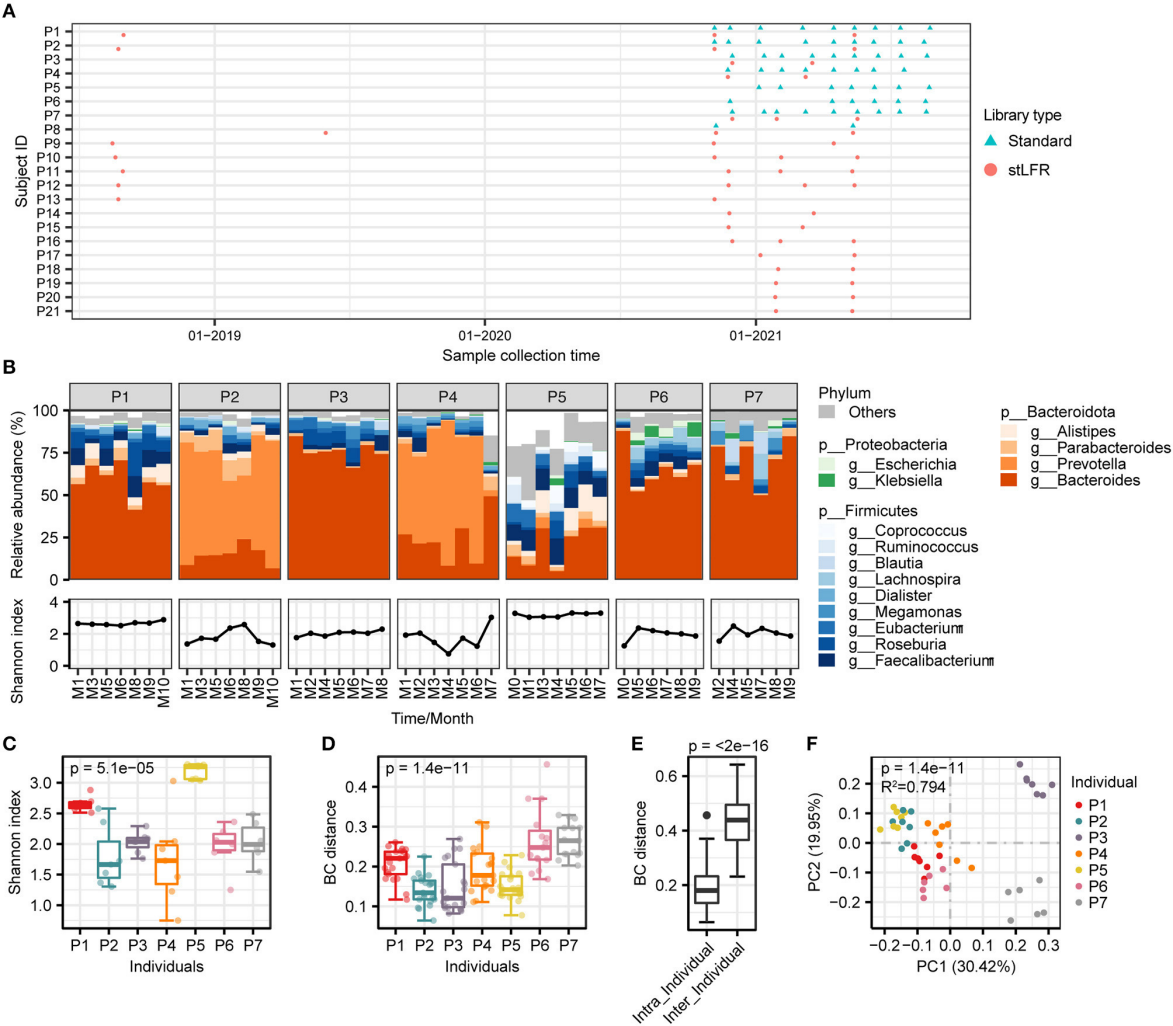
The composition and diversity analysis of gut microbiome in seven individuals. **(A)** Sampling collection of 96 samples from 21 individuals. The red circles represent samples of the stLFR library construction and the blue triangles for standard library construction. **(B)** Gut microbial composition. (Upper panel) the relative abundance of gut microbiome at the genus level of individuals over time; (Lower panel) alpha diversity of individuals over time. **(C, D)** The results of alpha diversity and beta diversity, and the Kruskal–Wallis test was used to determine significance. **(E)** Gut microbial composition was determined based on the Bray–Curtis distance, and the Wilcoxon rank-sum test was used to determine significance. **(F)** PCoA clustering analysis based on the Bray–Curtis distance matrix at the species level.

From November 2020 to August 2021, a total of 21 volunteers aged 23–42 years were recruited for this study (Table 1). Their stool samples were collected at intervals of 1–2 months by BGI, using the fecal sample collection kit (MCK-01 KMHD, Shenzhen, China), and were frozen at −80°C. In addition, seven stool samples collected in May 2018 and one stool sample collected in August 2019 were also included in this study, resulting in a total of 96 samples for sequencing analysis. For library construction, standard library construction was used for 46 samples, stLFR library construction was used for 37 samples, and the libraries of the other 13 samples were processed using both methods (Figure 1A). Detailed information on the dietary habits of all volunteers is collected and presented in Supplementary Table 1. According to self-reports, two individuals took antibiotics and 10 individuals took probiotics, while nine individuals experienced symptoms of diarrhea or constipation during sampling. All volunteers provided informed consent, and the study was approved by the Ethics Committee of BGI (BGI-IRB 20145).

**Table 1:** Demographic characteristics of the study.

**Characteristics**	***n* = 21**
Male, *n* (%)	14 (66.7%)
Age (mean ± S.D.)	29.65 ± 4.58
BMI (mean ± S.D.)	23.32 ± 3.78
stLFR library sequencing (individual/sample)	21/50
Standard library sequencing (individual/sample)	7/47
Antibiotic (individual/sample)	2/2
Probiotics (individual/sample)	10/21
Constipation/diarrhea (individual/sample)	9/24

### DNA extraction, library construction, and sequencing

For 59 samples collected at 1- to 2-month intervals of eight individuals, genomics DNA from 200 mg of stool samples was extracted using a MagPure Stool DNA KF Kit B (Magen, China), according to the manufacturer's instructions. The DNA was quantified with a Qubit Fluorometer using a Qubit dsDNA BR Assay Kit (Invitrogen, United States), and its quality was verified by running an aliquot on a 1% agarose gel. A total of 1 μg of genomic DNA was randomly fragmented by Covaris E220 (Covaris, Brighton, UK), and the resulting fragments with an average size of 200–400 bp were selected using magnetic beads. The selected fragments underwent end-repaired, 3' A-tailed, adapter addition, PCR amplification, and magnetic bead purification. The double-stranded PCR products were denatured by heating and circularized by splint oligo sequence. The single-stranded circular DNA (ssCir DNA) was formatted as the final library and sequenced on the MGISEQ-2000 platform (BGI, Shenzhen, China) with paired-end reads of 150 bp.

We selected 50 samples for stLFR library construction, which were obtained from 19 individuals. Details of stLFR library construction can be found in Wang et al. (2019). The qualified libraries were sequenced on the DNBSEQ-T1 platform (BGI, Shenzhen, China) using paired-end reads of either 100 bp (7 samples) or 150 bp (43 samples; Supplementary Table 2).

### Data quality control and microbiota diversity analysis from standard library construction

The raw reads from 59 samples were filtered using SOAPnuke v1.5.6 (Chen et al., 2018) with the following parameters: “-n 0.01 -q 0.4 -l 20 -d -Q 2 -G.” This step removed reads with low quality (Q20 <40%) and PCR duplicates and ambiguous base (N). Subsequently, the filtered reads were aligned to the human reference genome (GRCh38) using bowtie2 v2.3.4.3 (Langmead and Salzberg, 2012) to discard host DNA. As a result, a total of 960.72 Gb of data were obtained, with a data size per sample of 15.77 ± 3.62 Gb (mean ± S.D.). The average contamination rate of host DNA was found to be 0.32% (Supplementary Table 3). The species classification and relative abundance of each species in each sample were determined using MetaPhlAn3 v3.0.7 (Truong et al., 2017) based on quality-controlled reads. The within-sample species richness was estimated using the alpha diversity (Shannon index), while the dissimilarity between samples was evaluated using the beta diversity (Bray–Curtis) at the species level. Additionally, permutational multivariate analysis of variance (PERMANOVA) was performed using the Bray–Curtis distance, with 9,999 permutations.

### Assembly of sequencing data from the standard library

To investigate the impact of different library construction methods on assembly performance, a library comprising 13 samples was constructed using both the stLFR method and the standard method. For the standard method, high-quality reads were assembled using the *de novo* assembler MAGAHIT v1.2.9 (Li et al., 2015), with the parameters “–k-min 71 –k-max 81 –k-step 10,” and then binned using MetaBat 2 v2.15 (Kang et al., 2019). To evaluate the quality of the assembled genomes, CheckM v1.1.2 (Parks et al., 2015) software was used to classify them according to their completeness and contamination. Genomes were classified as low quality if completeness was ≤50% or contamination was ≥5%, medium quality if completeness was between 50 and 90% and contamination was <5%, and high quality if completeness was >90% and contamination was <5%.

### Assembly of sequencing data from the stLFR library and construction of reference genome catalog

The public script “stLFR_barcode_split” (Wang et al., 2019) was used to identify and remove barcode sequences in paired-end reads. To ensure the identification of reads from the same DNA fragment during assembly, barcode sequences in the read IDs of FASTQ files were replaced with numerical symbols using the same script. The raw data without barcode sequences were filtered using SOAPfilter v2.2 (Kar et al., 1996) with the following parameters: “-y -p -M 2 -f−1 -Q 10,” to discard low-quality, adaptor, and duplicated reads. A total of 3,649.41 Gb of high-quality data were obtained from 50 samples for further analysis, with an average sample size of 72.99 ± 17.68 Gb (mean ± S.D.). The high-quality reads were assembled using MetaTrass (Qi et al., 2022), developed for metagenomic stLFR library sequencing data, and the assembled genomes were quantified using CheckM v1.1.2 (Parks et al., 2015). To eliminate redundant high-quality genomes, dRep v3.2.0 (Olm et al., 2017) was employed to construct a catalog of reference genomes, with parameters set to “-pa 0.9 -sa 0.95 -nc 0.6 -cm larger.” Ribosomal RNAs (rRNAs) of the reference genomes were predicted using Barrnap v0.9 (default parameters) to demonstrate the precision of the assembled genomes. In addition, GTDB-Tk v2.1.1 (Parks et al., 2018; Chaumeil et al., 2020) was utilized to perform taxonomic annotation of the reference genomes based on the released annotation library release207_v2. Given the missing 16S rRNA in some genomes, a phylogenetic tree of the reference genomes was constructed using protein sequence alignments obtained by GTDB-Tk by IQ-TREE v1.6.6 (Nguyen et al., 2015), with parameters set to “-m LG+F+R10,” and visualized using Interactive Tree of Life (iTOL) v6 (Letunic and Bork, 2019; https://itol.embl.de/).

### Analysis of SNP of gut microbiota in individuals

The quality control sequencing reads of 96 samples were aligned to the constructed reference genomes using BWA v0.7.17, with default parameters. The coverage depth and breadth of each sample for each reference genome were calculated by an in-house script. Reference genomes that had at least 40% coverage in at least one sample and a cumulative depth of coverage of more than 10X for all samples were selected as references for SNP calling. A total of 302 species met these criteria. SNPs and INDELs were called using GATK v4.1.2.0 with the UnifiedGenotyper model. Only SNPs and INDELs that were present in at least two samples and were supported by at least four reads were retained. The obtained VCF file was used to calculate a pairwise p-distance matrix between samples using VCF2Dis v1.47 (https://github.com/BGI-shenzhen/VCF2Dis). A neighbor-joining tree was constructed using fneighbor (http://emboss.toulouse.inra.fr/cgi-bin/emboss/fneighbor?_pref_hide_optional=0), and the distance tree of samples was visualized using iTOL (Letunic and Bork, 2019).

### The estimation for π and FST values of nucleotide diversity

The π value of nucleotide diversity is a quantification indicator of genetic variation and can measure the degree of polymorphism in a population (Schloissnig et al., 2013). Equation 1 defines genetic variation as the average difference between corresponding regions of genomic DNA sampled randomly from individuals within a population.

The mean value of the difference between the same regions of genomic DNA sampled randomly from a sample (population) is defined by Equation 1 to estimate π in a metagenomic sample:


(1) $$\begin{array}{l} \pi \left(S,G\right)=\frac{1}{\left|G\right|}\overset{\left|G\right|}{\underset{i=1}{\sum }}\underset{B_{1}\epsilon \left\{ACTG\right\}}{\sum }\underset{B_{2}\epsilon \left\{ACTG\right\}\backslash B_{1}}{\sum }\frac{x_{i,B_{1}}}{c_{i}}\frac{x_{i,B_{2}}}{c_{i}-1} \end{array}$$

where *S* is the sample, *G* is the genome of interest, |*G*| is the size of the genome, *x*_*i,Bj*_ is the number of nucleotides *B*_*j*_ seen at position *i*, and *c*_*i*_ is the coverage at position *i* in the genome.

From the above definition, π between two samples is naturally defined by Equation 2:


(2) $$\begin{array}{l} \pi \left(S_{1},S_{2},G\right)=\frac{1}{\left|G\right|}\overset{\left|G\right|}{\underset{i=1}{\sum }}\underset{B_{1}\epsilon \left\{ACTG\right\}}{\sum }\underset{B_{2}\epsilon \left\{ACTG\right\}\backslash B_{1}}{\sum }\frac{x_{i,B_{1},S_{1}}}{c_{i,S_{1}}}\frac{x_{i,B_{2},S_{2}}}{c_{i,S_{2}}} \end{array}$$

where *x*_*i, Bj, Sk*_ is the number of nucleotides *B*_*j*_ seen at position *i* in the sample *S*_*k*_ and *c*_*i,Sk*_ is the coverage at position *i* in the sample *S*_*k*_ in the genome.

The fixation index (FST) is an indicator that measures the differentiation of the population, and the greater the FST, the greater the group difference (Schloissnig et al., 2013). FST is standardly defined by Equation 3:


(3) $$\begin{array}{l} F_{ST}\left(S_{1},S_{2},G\right)=1-\frac{\pi_{within}}{\pi_{between}}=1-\frac{\left(\pi \left(S_{1},G\right)+\pi \left(S_{2},G\right)\right)/2}{\pi \left(S_{1},S_{2},G\right)} \end{array}$$

where *F*_*ST*_ is commonly distributed at the interval [0,1], with close to zero indicating highly similar samples, and values around 1 indicating strong differentiation. Theoretically, negative values may occur and are often either interpreted as out-breeding or rounded to 0.

### Detection of horizontal gene transfer (HGT)

In this study, the detection of HGT of genomes from the stLFR library was performed by comparing the sample genome to reference genomes. First, quality control reads from the sample were aligned to reference genomes, and those with more than 40% coverage were selected for further HGT analysis. Two approaches were combined for HGT detection as follows: the best-match approach and phylogenetic incongruency using the metagenome HGT detection software MetaCHIP v1.10.10 (Song et al., 2019), with parameters “-r g -al 500 -cov 100.” The genetic distance between bacterial species where HGT occurred was calculated using FastANI v1.32 (Jain et al., 2018). The frequency of HGT in each individual was assessed by determining the proportion of HGT occurring per 100 pairs of species (Smillie et al., 2011).

### Annotating transferred genes

The transferred genes were initially annotated using eggNOG-mapperv2.1.2 (Cantalapiedra et al., 2021) and InterProScan v5.39-77 (Quevillon et al., 2005). To identify antibiotic resistance, virulence genes, and COG categories, Diamond v0.9.10.111 with an e-value of 1e-5 threshold and a minimum coverage of 40% was employed with the Comprehensive Antibiotic Resistance Database (v4.0; Alcock et al., 2020), virulence factor database (Liu et al., 2019), and COG database (v2014; Galperin et al., 2015).

The classification of antibiotic resistance genes and mobile elements was determined based on the resistance mechanism and relevant keywords. The mobile element classification was performed using keywords described in a previous study (Smillie et al., 2011):

Transposons: transpos^*^, TN, insertion element, is element, IS element;

Phage: phage, tail protein, tegument, capsid;

Plasmid: relaxase, conjugal transfer, Trb, relaxosome, type IV secretion, conjugation, Tra[A-Z], Mob[A-Z], Vir[A-Z][0-9], t4ss, T4SS, resolvase, antirestriction;

Other MGE: recombinase, integrase.

## Results

### Different gut microbial compositions in individuals

We selected 47 samples from seven individuals who underwent the standard library construction method to analyze the dynamic diversity of gut microbial composition over time. The results showed that the microbial composition of an individual remained similar over time, but there were significant differences across individuals (Figure 1B). Specifically, *Bacteroides* was more abundant in individuals P1, P3, P6, and P7, while *Prevotella* was higher in P2 and P4 individuals. In the case of individual P5, *Bacteroides, Faecalibacterium*, and *Alistipes* were the dominant species (Figure 1B). These differences in microbial composition between individuals may contribute to the dominance of *Bacteroides* and *Prevotella* as enterotypes. The species richness at different times within an individual was fluctuating in some persons (Figure 1B) and was significantly different between individuals (Kruskal–Wallis test, *p* = 5.1e-05, Figure 1C). Beta diversity analysis showed that the microbial composition was more similar within an individual than between individuals (refer to Figures 1D–F), consistent with the conclusions reported previously (Xie et al., 2016).

### Comparison of assembly results between stLFR and standard libraries

**Figure 2:**
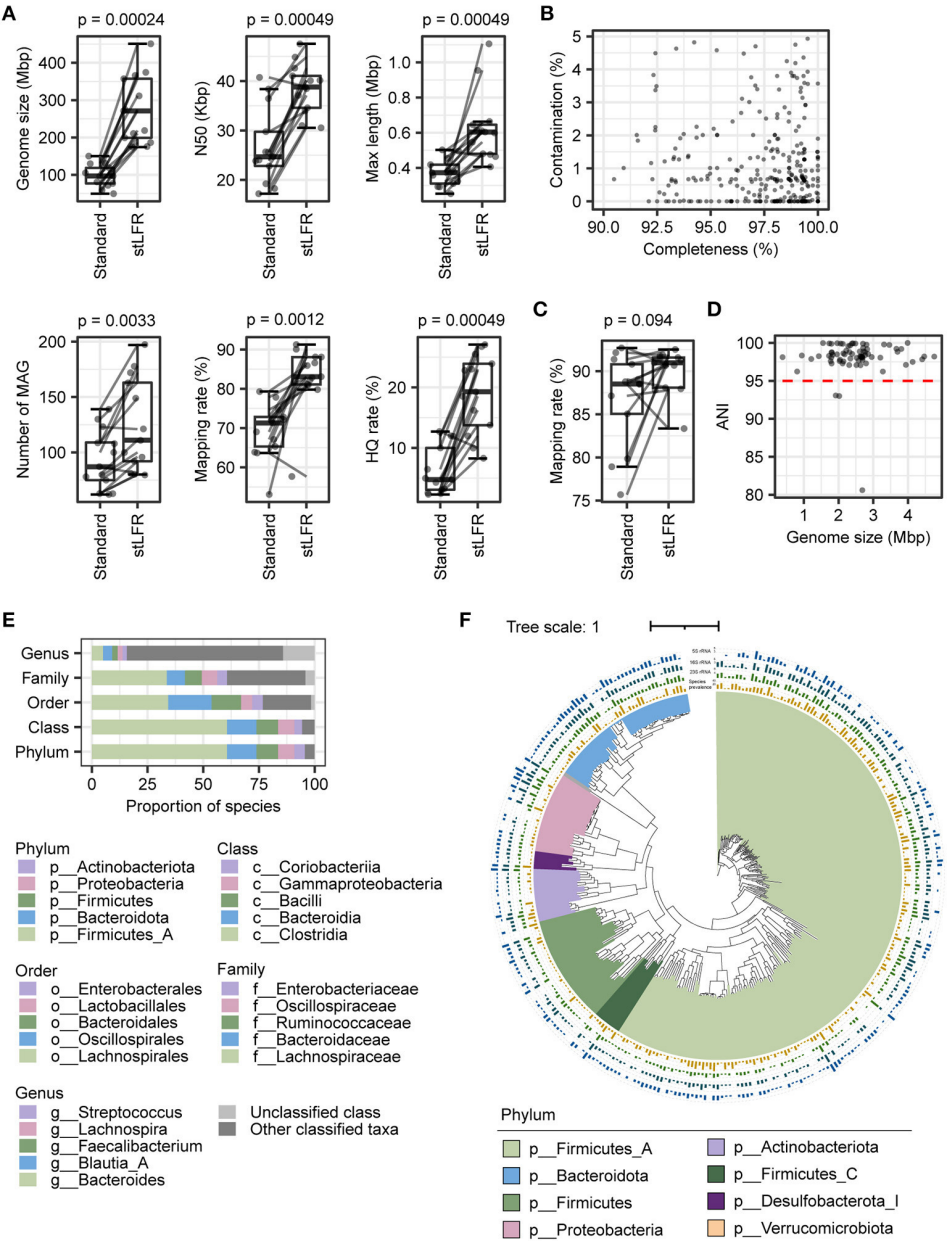
Comparison of assembly results of the stLFR and standard methods and construction of reference genomes catalog. **(A)** Assembly assessment metrics for samples from 13 individuals were processed using both the stLFR and the standard methods, respectively. Wilcoxon rank-sum test was used to determine significance. **(B)** Completeness and contamination scores for each of the 318 MAGs with high quality. **(C)** The mapping rate to 318 MAGs for samples from 13 individuals was processed using both the stLFR and standard methods, respectively. Wilcoxon rank-sum test was used to determine significance. **(D)** ANI between 318 MAGs and high-quality MAGs obtained from the standard method. **(E)** Taxonomy composition of the 318 MAGs at phylum, class, order, family, and genus levels. **(F)** Phylogenetic tree of the 318 reference genomes. The yellow bars in the innermost layer represent the prevalence of the genome among the samples (coverage breadth >40%). The green, dark green, and blue bars represent the number of 5S, 16S, and 23S rRNAs predicted in the reference genomes, respectively.

To evaluate the impact of two library construction methods on metagenome assembly, we selected the assembly results of 13 paired samples that were processed using both the standard method and stLFR method simultaneously and performed a paired Wilcoxon rank-sum test analysis on them. A total of 1,217 and 1,620 metagenome-assembled genomes (MAGs; length > 0.2 Mb) were produced by the standard method and stLFR method, respectively. The vast majority of standard and stLFR MAGs were at least of low quality (Supplementary Figures 1A, B). By comparing assembly properties, we found that the median of genome size (*p* = 2.4e-04), N50 (*p* = 2.4e-04), contig maximum length (*p* = 4.9e-04), and number of MAGs (*p* = 3.3e-03) significantly increased with the stLFR method (Figure 2A and Supplementary Tables 2, 3). Although the stFLR method results in a high contamination rate (Supplementary Figure 1B), the proportion of high-quality MAGs generated with stLFR (19.01 ± 6.48%) was significantly higher than that with the standard method (6.21 ± 3.99%; *p* = 4.9e-04). Additionally, the mapping rate of MAGs increased from 69.91 ± 7.38% using the standard method to 82.89 ± 8.43% using the stLFR method (*p* = 1.2e-03, Figure 2A and Supplementary Tables 2, 3). These results suggest that the stLFR method is a superior approach for metagenome assembly compared to the standard method.

### Construction of reference genomes using assembly genomes generated by the stLFR library

Previously, the superiority of the stLFR method in the metagenomic assembly has been demonstrated, which is consistent with our results. Therefore, we utilized the stLFR method to process 50 samples and constructed a reference genome catalog comprising 6,844 MAGs (length ≥ 0.2M), including 1,388 high-quality, 911 medium-quality, and 4,545 low-quality MAGs (Supplementary Figure 1C). Subsequently, we performed de-redundancy on the high-quality MAGs, using dRep to obtain 318 non-redundant MAGs ranging from 1,153,651 to 6,730,161 bp in size (Figure 2B and Supplementary Table 4). We analyzed the read mapping rates to 318 MAGs of 13 samples with both the standard and stLFR methods. The read mapping rate for the 13 standard library samples was 82.89 ± 8.43%, while that for the 50 stLFR library samples was 89.37 ± 4.51%, showing an improvement in comparison to the read mapping rates of their respective self-assembled results (Figures 1A, 2C, and Supplementary Tables 2, 3). The ANI index of 73 high-quality MAGs obtained by standard methods, as well as the low- and medium-quality MAGs from the stLFR method, concerning de-redundant MAGs, suggested that the 318 MAGs represented most of the MAGs obtained from the standard and stLFR methods (Figure 2D and Supplementary Figure 1D). The dominant phyla of the 318 MAGs were Firmicutes_A (60.70%), Bacteroidota (13.21%), Firmicutes (9.75%), Proteobacteria (7.23%), and Actinobacteriota (4.72%; Figure 2E and Supplementary Table 4). Furthermore, a phylogenetic tree of 120 proteins from the samples extracted by GTDB-TK was constructed by iqtree software, and we found 120 types of proteins that covered multiple different phyla at the phylum level (Figure 2F). In addition, the 5S, 16S, and 23S rRNA of 235 MAGs (73.90%) were identified successfully. Among 318 MAGs, there are only 12 MAGs, each of which was only detected in one sample and the rest were present in at least two samples, and more than half were identified in samples above 10 (Figure 2F). These results indicate that the 318 MAGs generated from stLFR libraries were relatively complete genomes, covering a large portion of the gut microbiome in the samples. Overall, the reference genomes were successfully constructed based on the stLFR method and were proposed for use as a reference for intestinal flora analysis.

### Nucleotide diversity quantification analysis of the gut microbiome

**Figure 3:**
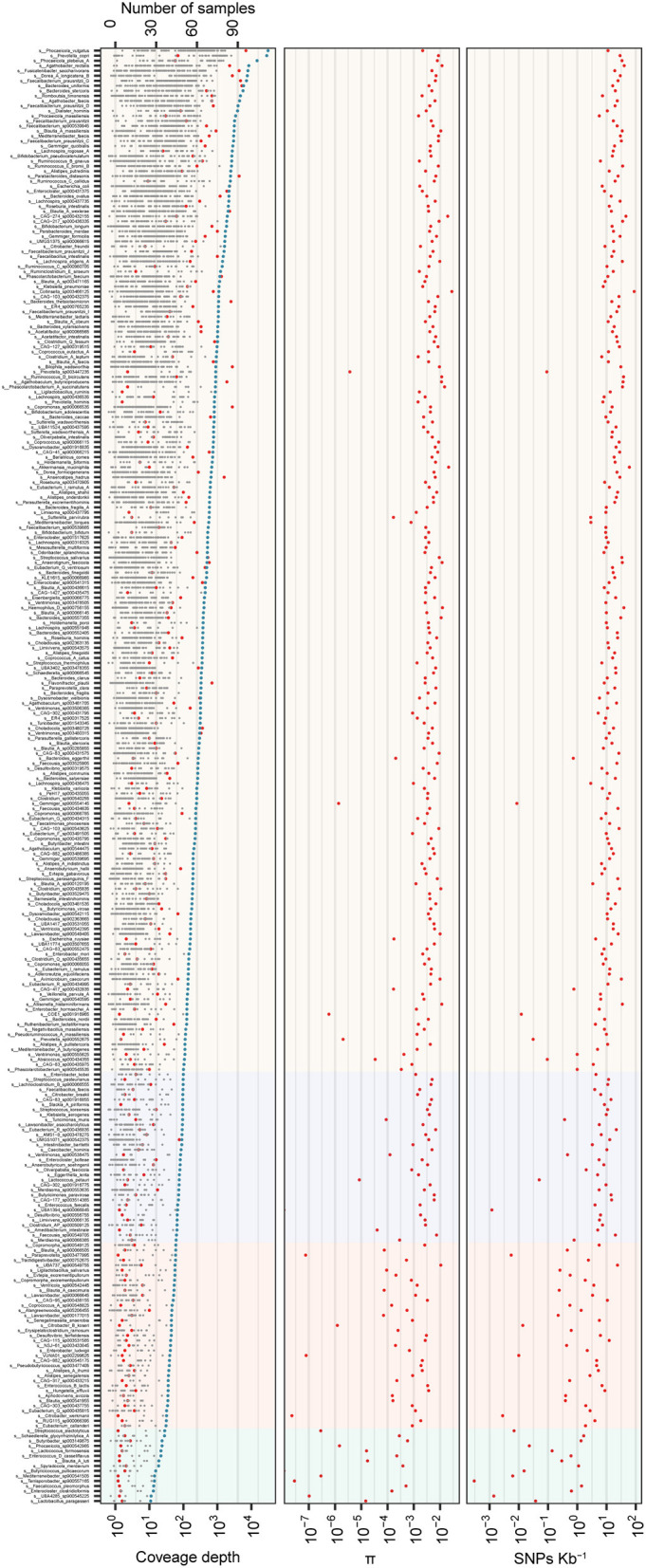
Genome variation of 290 MAGs in 96 samples from 21 individuals. In total, 290 MAGs with coverage breadth of ≥ 40% and cumulative coverage depth of ≥ 10X were shown in the figure. **(Upper panel)** the gray point indicated the coverage depth of the sample, the blue points indicated the cumulative coverage depth, and the red points represented the prevalence of species; **(Middle panel)** a box plot of strains diversity of samples; **(Lower panel)** SNPs frequency of strains. The light background represents 10–30X, 30–60X, 60–100X, and >100X coverage depth from left to right.

To illustrate the ability of assembled reference genomes by the stLFR method for intestinal flora analysis and know about nucleotide diversity of the gut microbiome, SNP and INDEL analyses were performed for investigating the nucleotide diversity of strain from gut microbiome within and between individuals based on 318 MAGs. According to the filter criteria, 302 species were selected as reference, and 11,066,694 SNPs and 473,831 INDELs were detected in 290 species and 282 species, respectively, from 96 samples (Supplementary Figure 2 and Supplementary Table 4). The distribution of SNPs per species ranged from 1 to 180,429, while INDELs ranged from 1 to 8,623 (Supplementary Figure 2 and Supplementary Table 4). Further analysis revealed that some species within Firmicutes_A and Bacteroidota had a particularly high frequency of SNPs, while other phyla had fewer SNPs (Supplementary Figure 2). Most species exhibited more than one SNP per 1,000 bases, with an increase in SNP number as coverage depth increased (Supplementary Figure 2). Then, the index π was used for the qualitative analysis of the polymorphism of the gut microbiota, and the distribution trend of nucleotide diversity of different species (π) demonstrated that the higher the nucleotide diversity of a species, the greater the diversity of the species population (Figure 3). As the coverage depth of samples increases, the number of SNPs also increased, with stability observed at a coverage depth of 60X (Figure 3). This finding suggests that a coverage depth of 60X is sufficient for the stLFR method.

### Comparison of polymorphisms across different individuals

**Figure 4:**
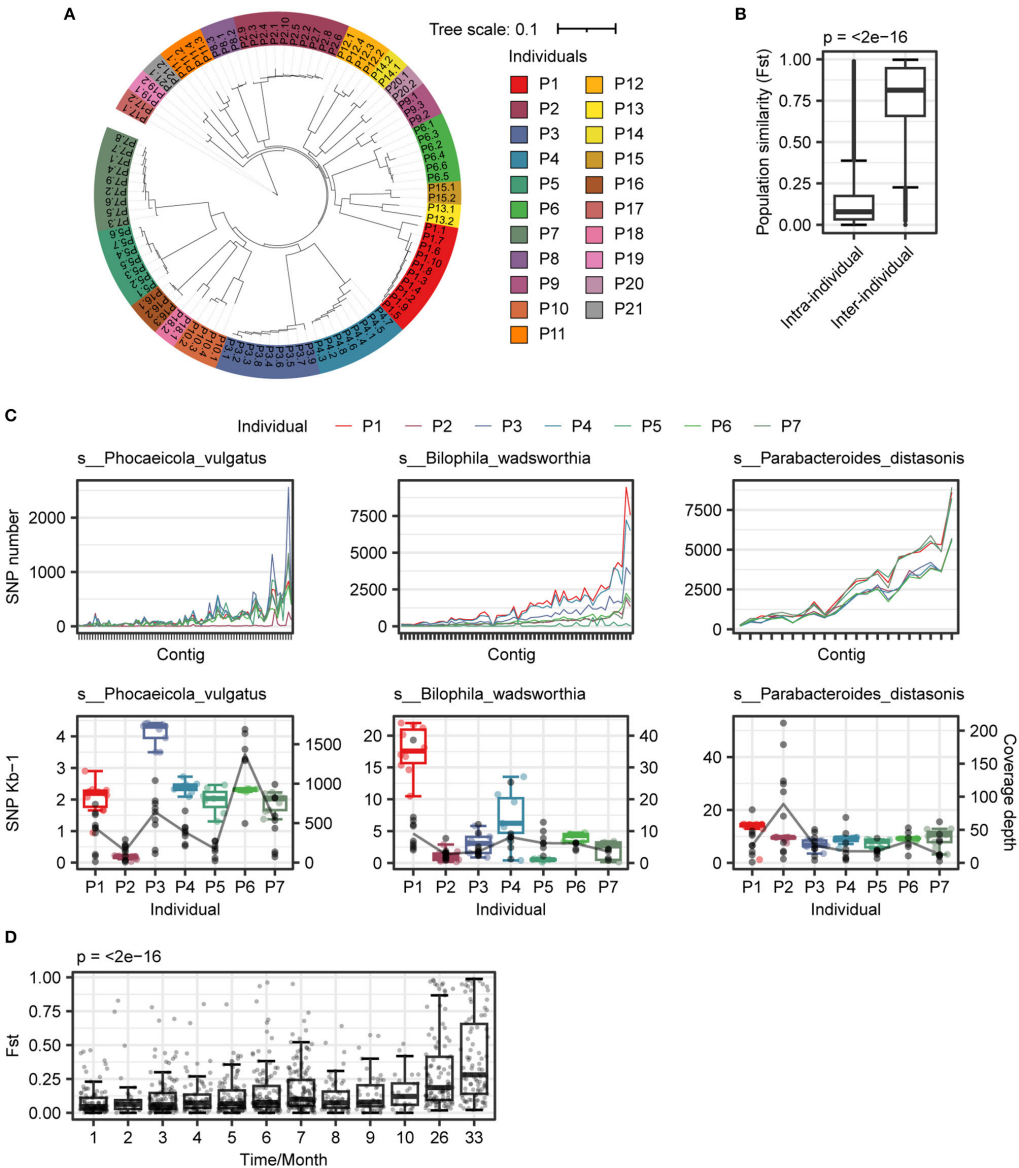
Analysis of genome variation intra- and inter-individuals. **(A)** Construction of distance tree based on SNPs p-distance between samples. The same color indicated samples from the same individual. **(B)** The variability of strains in intra-individuals was less than that in inter-individuals. **(C)** Polymorphism patterns of three species in individuals. (Upper panel) genome-wide patterns of SNPs of three species in individuals; (Lower panel) SNPs frequency (box plot) and coverage depth (black point) of three species in individuals. **(D)** Diversity dynamic trace within strain in individual. A total of seven individuals (P1–P7) and eight individuals (P1, P2, and P8–P13) were selected for Fst calculation within (1–10 months) and more than a year (26 and 33 months), respectively. Wilcoxon rank-sum test and Kruskal–Wallis test were used to determine significance for two and more than two groups, respectively.

After comparing the SNP difference at the species level, we analyzed the variation in SNPs and INDELs within different individuals. The number of SNPs and INDELs among different individuals was significantly different, and the P5 individual has the highest diversity with the largest number of SNPs and INDELs (Supplementary Figure 3). The SNP distance tree showed that bacterial strains from the same individual were more similar (Figure 4A). The population diversity index Fst also showed that the diversity of strains within individuals was lower (Figure 4B), consistent with the results of individual community structure (Figure 1E).

To investigate the mutation patterns that contribute to causing species variation and similarity, we selected three different species for further analysis. We found that there were distinct mutation patterns in different individuals of each species (Figure 4C). We also examined the influence of time on species diversity within individuals. As depicted in Figure 4D, the range of changes in species diversity was not substantial within a 10-month period. However, after more than 24 months, there was a significant change in species diversity within individuals (*p* < 0.001). The similarity of strains within individuals decreased over time, and after accumulating to a certain extent, it led to significant changes in species diversity.

### The analysis of HGTs

**Figure 5:**
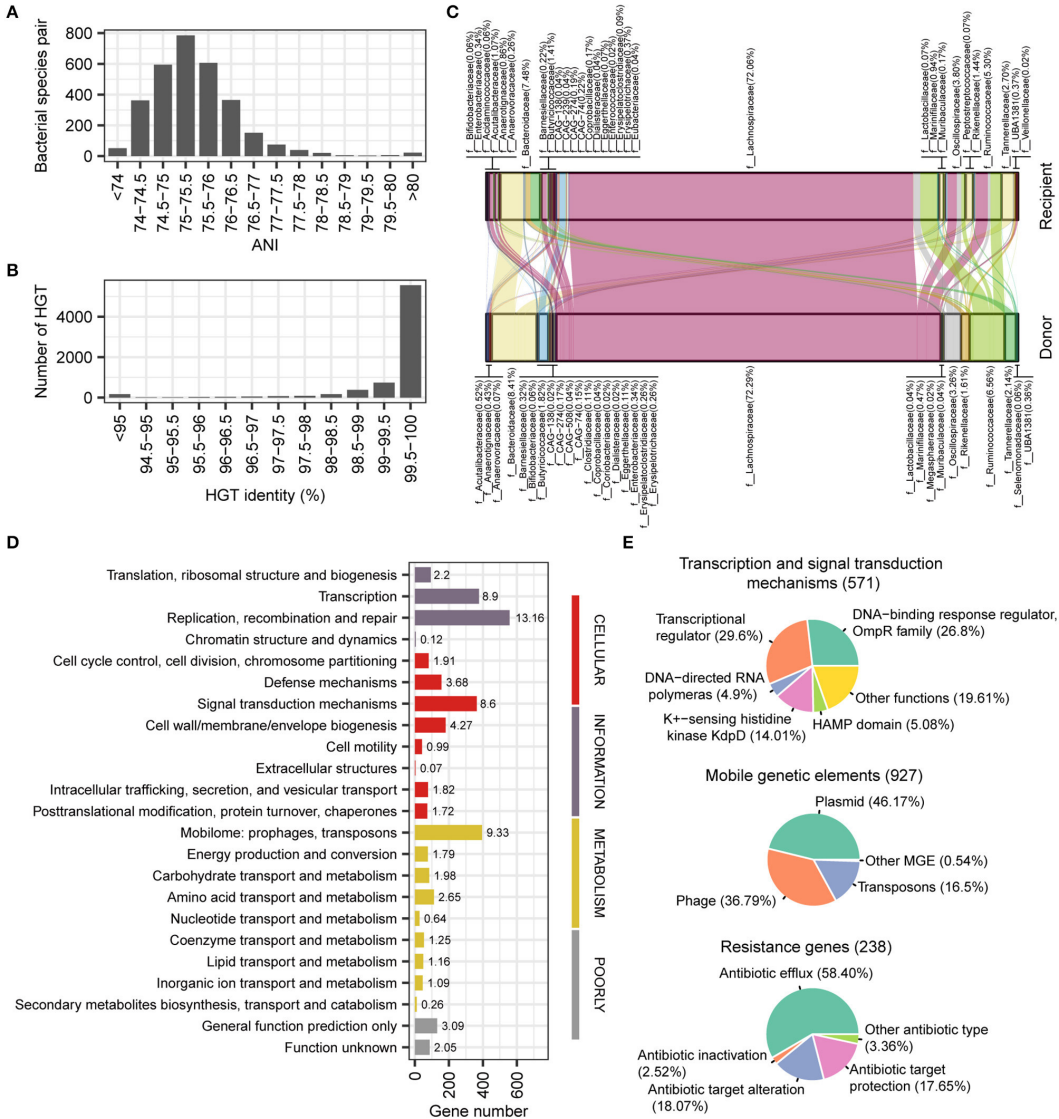
Detection and function of horizontal transferred genes. **(A)** Phylogenetic distance between two species occurred horizontal transferred gene. **(B)** Gene identity between horizontal transferred genes. **(C)** Predicted gene flow of horizontal transferred gene from donor species to recipient species. **(D)** COG category classification of horizontal transferred genes. **(E)** Main function classification of transcription and signal transduction mechanisms, mobile elements, and resistance genes.

Horizontal gene transfer (HGT) plays an important role in the human gut microbial ecosystem and is an important way of resistance gene transfer. The stLFR method has an advantage for metagenomic assembly and also facilitates the identification of HGT. Based on the reference assembled genome, we conducted an HGT analysis of 50 samples precessed by the stLFR method, resulting in a total of 7,338 HGTs involving 3,842 unique genes (335 species; see Supplementary Table 5). According to the ANI index, it appeared that horizontal gene transfer (HGT) did not occur within species (ANI > 97%). Instead, HGTs mainly occurred at levels above the genus (ANI < 80%) and were concentrated between 75 and 76% (Figure 5A). Furthermore, we found that 6,308 out of the 7,338 HGTs detected had a similarity identity of over 99% (Figure 5B), indicating that the majority of the HGT events in gut microbiota were recent transfer events. The families with the most frequent occurrence of HGT were Lachnospiraceae, Bacteroidaceae, Ruminococcaceae, and Oscillospiraceae (Figure 5C). We found that the three functions that contain the most HGTs are replication, recombination, and repair; mobilome prophages; transposons and transcription, respectively (Figure 5D). For transcription and signal transduction mechanism genes, OmpR family and transcriptional regulator account for more than 50% (Figure 5E). For mobile elements genes, there are mainly three types of plasmid (46.17%), phage (36.79%), and transposons (16.50%), and we also found some antibiotic resistance genes relating to antibiotic efflux (58.40%), antibiotic target protection (17.65%), and antibiotic target alteration (18.07%; Figure 5E).

From the perspective of the ANI index, it appears that horizontal gene transfer (HGT) does not occur within species (ANI > 97%). Instead, HGT mainly occurs at levels above the genus (ANI < 80%) and is concentrated between 75 and 76% (Figure 5A). Despite this, the similarity of genes remains very high (identity > 99%; Figure 5B), suggesting that most HGTs are recent transfer events in the gut microbiota. Overall, these findings suggest that HGT plays an important role in shaping the genetic diversity of the gut microbiota across different taxa.

**Figure 6:**
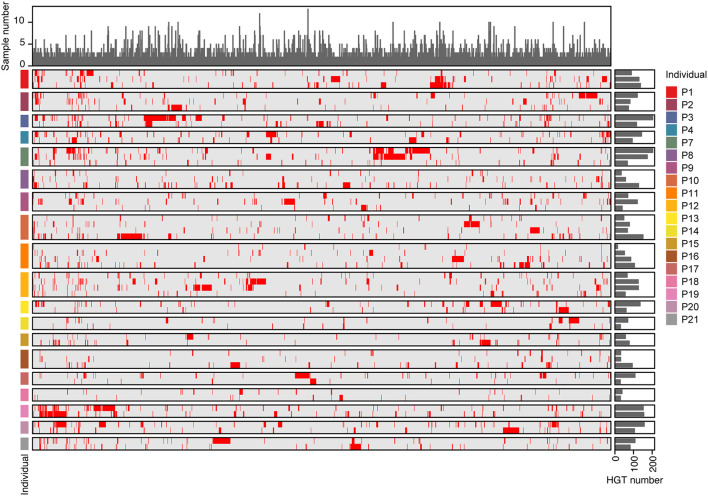
The distribution of HGTs in individuals. The rows of the heat map represented different samples, while the columns represented the 1,496 HGTs events that were detected in at least two samples. Red in the heat map indicated that HGTs were detected in the samples, while gray indicated their absence. Bar plots on the right side of the heat map showed the number of HGTs detected in each sample. Bar plots on the top showed the number of samples where each HGT was detected.

We observed the incidence of HGT across samples, and the number of HGTs in individuals ranged from 26 to 315 (Supplementary Table 5). Of the 3,842 distinct HGTs, only 1,497 were found in more than two samples at the same time. Furthermore, only 96 HGTs were detected in at least one individual across all time points (Figure 6 and Supplementary Table 5). There was no obvious variation in the number of HGT samples at different time points in the same individual. These results indicate that most HGT events are specific to the sample.

## Discussion

In this study, we aimed to explore the potential of the stLFR method for application in metagenomics by comparing assembly results with those of the standard method. The comparison results showed that the stLFR method achieved better assembly indicators, including genome length, contig maximum length, NG50 length, and high-quality MAGs, which is consistent with the results of the 10x linked-read-based study (Siranosian et al., 2022). Using the stLFR method, we constructed a reference genome catalog containing 318 MAGs from 50 samples. Due to the number of samples, we obtained fewer non-redundant MAGs than UHGG (4,644 MAGs) and 10x linked-reads (1,615 MAGs). However, in terms of MAG completeness, 73.90% of the MAGs in our genome catalog contain 5S, 16S, and 23S rRNA, while in UHGG and 10X linked-reads, the percentages are 12.30 and 27.00%, respectively. These results illustrated that our genome set is more complete (Almeida et al., 2021; Siranosian et al., 2022). In addition, the mapping rate of reads to this reference genome catalog was higher for both the standard and stLFR sequencing data, indicating that this genome catalog contains most of the species detected by both the standard and stLFR methods. The above results indicate that the stLFR method can be applied to the metagenomics study and can obtain a high-quality reference genome catalog.

TGS has been widely applied, not only in animals and plants but also in the field of metagenomics. It has achieved great success in improving genome assembly and detecting structural variations (Chen et al., 2022; Kim et al., 2022; Zhao et al., 2022). Although we did not compare the assembly results between the stLFR method and TGS method in this study, previous reports have shown the advantages of the stLFR method over TGS in the application of metagenomics. Compared with ONT, the stLFR method generated more species with an NG50 of around 2M and fewer mismatches and INDELs (Qi et al., 2022). Zhang demonstrated that the assembly results based on stLFR data generated more near-complete metagenome-assembled genomes than PacBio long-reads in real fecal samples (Zhang et al., 2022). These results show the potential of the stLFR method for metagenomics. However, this technique has some limitations. First, metagenomic studies involve multiple species in a community with varying abundance and DNA content in the sample, which may result in lower capture efficiency for low-abundance species. Despite increasing sequencing depth, we cannot guarantee that all species in the community will be included. Second, this method introduces barcodes to identify fragment origins, which requires systematic evaluation of its impact on species composition.

In a previous report, 10.3 million SNPs and 107,991 INDELs were identified in 101 genomes across 252 samples from 207 subjects (Schloissnig et al., 2013). In this study, using 96 samples from 21 individuals, we confirmed ~11.1 million SNPs and 473,831 INDELs, indicating that our method is capable of detecting more SNPs and INDELs due to the use of more complete genome assemblies. However, for SNP and INDEL analysis, the same conclusions were obtained, including there are significant differences in the number of SNPs and INDELs among different individuals, the individual has its unique species variation spectrum, and the similarity of strains within individuals decreased over time.

It is well-known that SNPs identification of the standard library construction method is severely affected by coverage depth (Schloissnig et al., 2013). Hence, we found that the coverage depth also has an effect on the SNPs identification of the stLFR method after analysis, but the 60X coverage depth is sufficient for this method. Therefore, the construction of the stLFR library is beneficial to the analysis of intestinal flora.

Furthermore, HGT analysis demonstrated that the HGT occurs mainly above the genus level, rarely within species due to poor assembly, and these HGT genes were similar (identity > 99%). Some of the identified HGT genes were related to antibiotic resistance genes, and these genes contributed to the development of antibiotic resistance of strains. In addition, it has been reported in the literature that in samples of the same individual at different time points, HGT may be retained over time, or new HGT may be added or lost (Groussin et al., 2021), which is consistent with our results. We also found that gender was revealed to not affect the incidence of HGT, and this may be because the living environment has not changed during this time.

## Conclusion

In conclusion, the stLFR library construction method was used to investigate the gut flora of humans and proved to be more advantageous for assembly metagenome and gut microbiota analysis. The reference genome constructed by stLFR can identify the SNPs, INDELs, and HGT genes efficiently.

## Data availability statement

The datasets presented in this study can be found in online repositories. The names of the repository/repositories and accession number(s) can be found at: CNGB Sequence Archive (CNSA) of China National GeneBank DataBase (CNGBdb) with accession number CNP0003344.

## Ethics statement

The studies involving human participants were reviewed and approved by the Medical Ethics Committee of BGI (BGI-IRB 20145). The patients/participants provided their written informed consent to participate in this study.

## Author contributions

XF, CG, ZY, and XX designed the study. YH, PJ, and ZL analyzed the results. CG, YH, and PJ wrote the manuscript. XF, RC, and ZY revised the study and supported the project. All authors reviewed the manuscript. All authors contributed to the article and approved the submitted version.
