# Supplementary material for: Assembly and analytical validation of a metagenomic reference catalog of human gut microbiota based on co-barcoding sequencing
Source: Front Microbiol. 2023 May 5;14:1145315. doi: 10.3389/fmicb.2023.1145315 (PMC10196144; doi:10.3389/fmicb.2023.1145315)
Supplement: Supplementary file 2 [file Data_Sheet_1.docx]

Suppermently figures


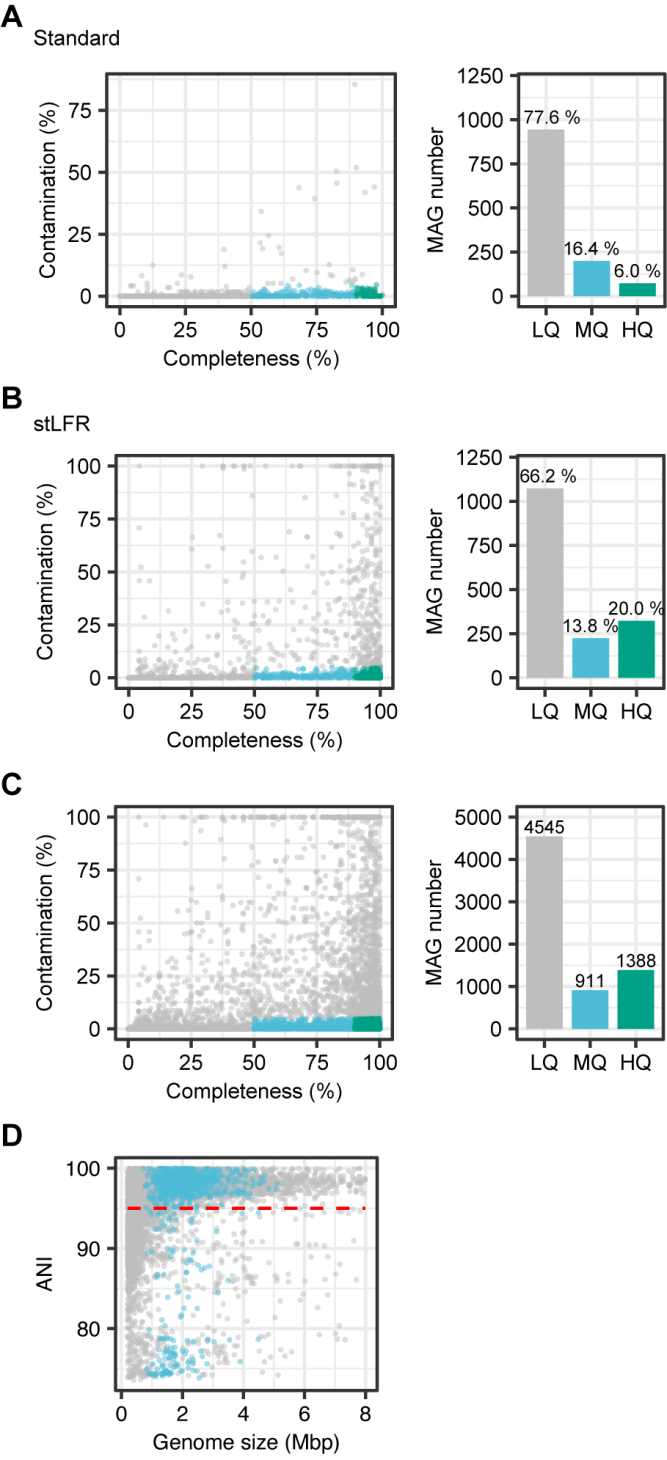


**Fig. S1 Quality assessment of MAGs. (A, B) Quality evaluation of 1,217 and 1,620 MAGs obtained from the standard and stLFR methods, respectively.** (C) Quality evaluation of 6,844 MAGs obtained from 50 samples using the stLFR methods. Dot plots were the completeness and contamination scores for MAGs, and colored by their quality classification category. Gray, low quality; Blue, medium quality; Geen, high quality. Bar plots were the number MAG according to the quality classification. (D) ANI for optimal alignment of low- and medium-quality MAGs to the 318 MAGs.


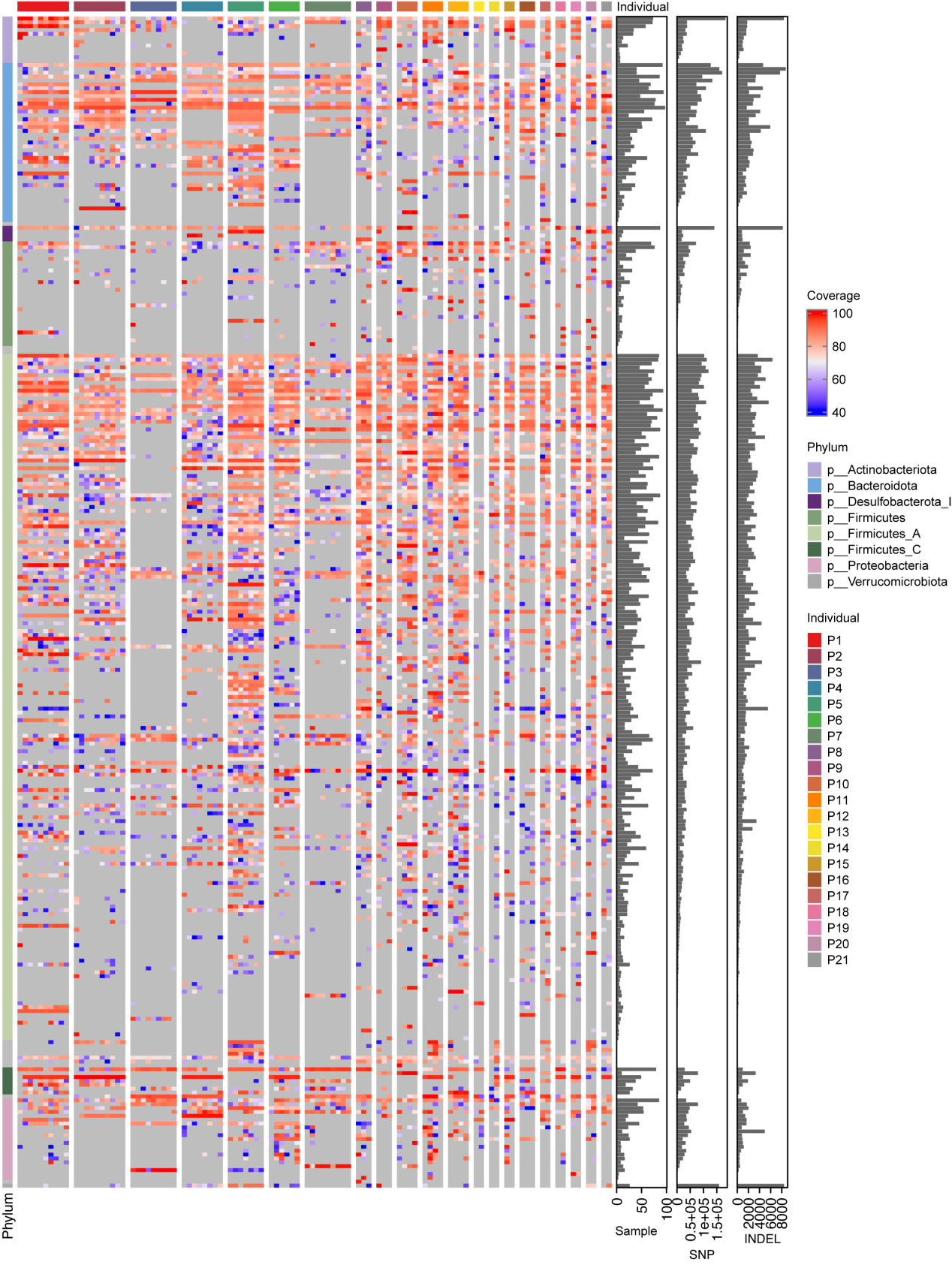


**Fig. S2 The coverage depth of samples to reference genomes and the number of SNPs and INDELs in species.** Heat map indicated the coverage of samples to 318 MAGs, where the gray color represented the missing of the MAGs in individuals. Row color bars indicated the the phylum to which the species belonged, while the column color bars indicated the individuals. Bar plots on the right side of the heat represented the number of samples, SNPs, and INDELs, respectively.


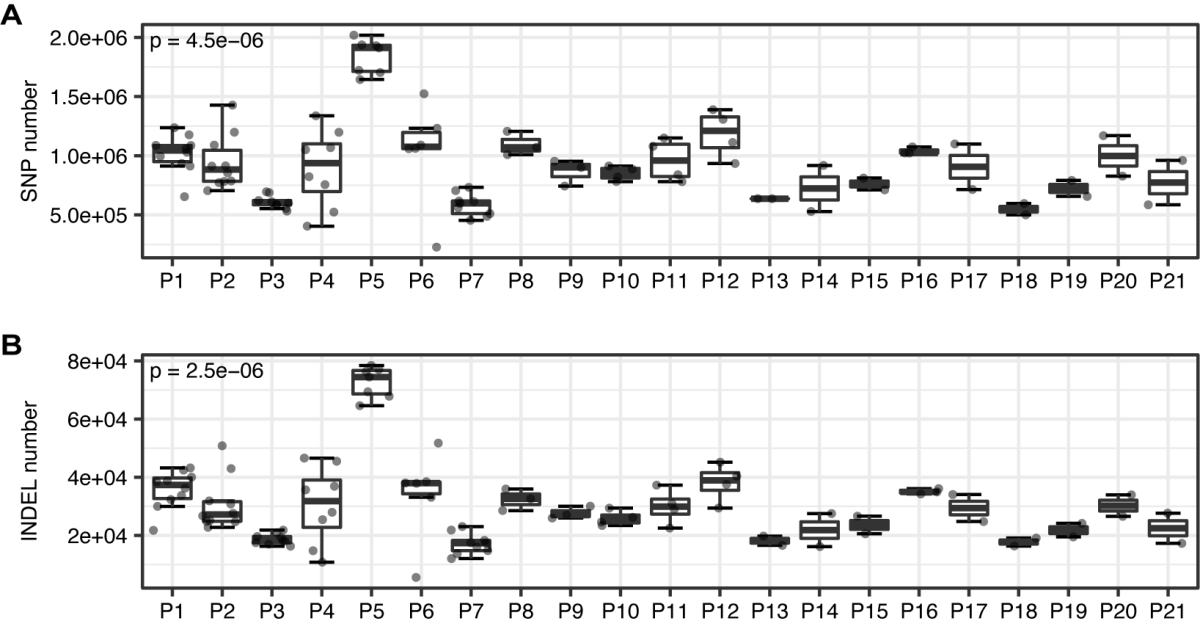


**Fig. S3 The distribution of SNPs (A) and INDELs (B) in 21 individuals.** Kruskal-Wallis test was used to determine significance between individuals.
